# Contemporary Use of Common Procedural Terminology (CPT) Codes for Surgical Management of Cerebellopontine Angle Tumors

**DOI:** 10.1055/a-2790-6085

**Published:** 2026-05-11

**Authors:** Shawn Stevens, Seilesh Babu, Kuhn Jeffery, Christine Dinh, Yuri Agrawal, Michael J. Link, J. Thomas Roland Jr, Colin L.W. Driscoll

**Affiliations:** 1 115467 Department of Otolaryngology, Barrow Neurological Institute, Phoenix, ArizonaUnited States; 2 Department of Neurotology, Michigan Ear Institute, Farmington Hills, MichiganUnited States; 3 Advent Health Group, Orlando, FloridaUnited States; 4 Department of Otolaryngology, University of Miami, Miami, FloridaUnited States; 5 Otolaryngology, University of Colorado Anschutz Medical Campus, Aurora, ColoradoUnited States; 6 Department of Neurologic Surgery, Mayo Clinic, Rochester, MinnesotaUnited States; 7 12297 Neurosurgery, New York University Medical Center, New York, New YorkUnited States; 8 12297 Otolaryngology, New York University Medical Center, New York, New YorkUnited States; 9 Department of Otorhinolaryngology, Mayo Clinic, Rochester, MinnesotaUnited States

**Keywords:** vestibular schwannoma, acoustic neuroma, cerebellopontine angle, common procedural terminology, CPT code

## Abstract

**Objectives:**

Cerebellopontine angle (CPA) tumors, including vestibular schwannomas (VS), often require microsurgical resection. These are typically surgical procedures performed by co-surgeons, and some controversy and confusion exist regarding use of common procedural terminology (CPT) codes for billing, given the American Medical Association (AMA) describes two distinct coding sets for such cases. This investigation endeavored to: (1) Comprehensively review all surgical coding options; (2) instruct on application; and (3) explore contemporary coding trends among surgeons.

**Design:**

A multi-disciplinary task force (otologist/neurotologists and neurosurgeons) performed direct correspondence with various surgical societies, private consultancies, and the AMA. Survey instruments were distributed to active surgeons.

**Setting:**

Investigational period: 2022 to 2025. Survey instruments distributed at the 2023 American Neurotology Society (ANS) Fall Meeting.

**Participants:**

ANS, American Academy of Otolaryngology (AAO-HNSF), and the North American Skull Base Society (NASBS) representatives.

**Outcomes Measures:**

Usage rate for CPT code sets (percentage of responders).

**Results:**

Two CPT coding systems are currently accepted for surgical management of CPA tumors. The traditional/legacy codes (Indicator-2) allow use of co-surgery modifiers (-62). The newer approach/resection codes (skull base codes; Indicator-1) restrict modifier use. The history, application, and controversies surrounding the codes are detailed herein. About 40% of surgeons reported use of the traditional codes, while 45 to 49% use the skull base codes. Response rate was 31% of the active ANS membership in 2023.

**Conclusion:**

The AMA recognizes two CPT code sets for surgical management of CPA tumors. Both are appropriate, if properly applied. Contemporary surgeons likely utilize the sets in equal proportions.

## Introduction

The cerebellopontine angle (CPA) is a complex three-dimensional space within the posterior cranial fossa on each side of the skull. The CPA contains key neurovascular structures including cranial nerves IV to XII and is bound by the brainstem and cerebellar hemisphere medially with the temporal bone forming the lateral boundary. The region is referred to as the lateral “skull base.”

Tumors of the CPA and lateral skull base are encountered at an incidence of between 1:50,000 and 1:100,000 and may require surgical intervention. The most common CPA tumor is the vestibular schwannoma (VS; also called acoustic neuroma). These benign neoplasms of the 8th cranial nerve account for roughly 80% of all tumors in this region. The next most common tumors encountered are meningiomas, with other benign and malignant tumor types occurring only rarely.

For surgeons to safely treat tumors in this region, an “approach” is required that can maximize exposure of the tumor while minimizing disruption to the surrounding anatomy and limiting brain retraction. The “approach” allows the highly skilled micro-surgical “resection” of the tumor, preserving neurovascular structures within the CPA, if possible. Given the complexity of this anatomical region and the tumors that occupy it, operations (surgical procedures) may become long and technically challenging.

For many advantageous reasons, most tertiary care centers now employ a dual (co-)surgeon team for surgical management of CPA tumors (especially VS). This dual team is comprised of a neurotologist (board sub-certified otolaryngologist with lateral skull base training) and a neurosurgeon. Tumors may also be managed by either specialty individually in select cases. Co-surgical management of CPA tumors has been associated with excellent outcomes, low morbidity, and very low to 0% mortality rates. 1 2 3 4

The most common skull base surgical approaches designed to treat CPA tumors include the translabyrinthine approach, the retrosigmoid approach, and the middle cranial fossa approach. Surgeons select a given approach based on a variety of tumor and patient-specific factors, as well as comfort level of the surgical team. At the conclusion of these procedures, participating surgeons submit common procedural terminology (CPT) codes that reflect the nature of the operation. These are then processed by professional coding teams to assess for accuracy and for appropriate supporting documentation, after which the filings transition to billing teams to submit for financial compensation.

In the case of surgery for CPA tumors, surgeons have multiple CPT code sets to choose from. The differing code sets, in general terms, have been dubbed the “traditional/legacy” codes (combined code for approach/resection) and the “skull base” codes (split approach and resection codes). At the time of this writing, both coding sets are recognized by the American Medical Association (AMA) CPT Editorial Panel, the relevant CPT Advisory Panels, and the Centers for Medicare/Medicaid Services (CMS). 5 6 7 8 9 10 11 12

Recently, however, confusion and controversy have arisen regarding proper use of these CPT codes, especially for management of VS tumors. 9 10 13 14 In response to this, a multi-disciplinary task force was assembled with the support of four professional surgical societies to investigate and address this controversy. The objectives of this effort were to: (1) Define current CPT coding options for the surgical management of CPA tumors, (2) construct a reference to guide surgeons and their coding teams managing CPA tumors, and (3) report on current trends in the utilization of both the traditional/legacy and skull base code sets among surgeons (ca. 2023).

## Methods

### Timeline

This investigation was conducted longitudinally from September 2022 to September 2025.

### Task Force

A multi-disciplinary task force was convened to include both neurotologists and neurosurgeons. This included representatives of the American Neurotology Society (ANS – Education/Socioeconomic/Executive Committees), American Academy of Otolaryngology – Head and Neck Surgery (AAO-HNS; Skull Base Surgery Committee), and the North American Skull Base Society (NASBS). Direct correspondence was also conducted with the American Academy of Neurological Surgery (AANS) as well as a third-party consultancy, experienced with surgical coding practice for CPA tumors. 6

### Coding Descriptors, Definitions, and Authoritative Citation

Definitions and descriptors of current CPT codes, modifiers, designations, and categories were obtained through reference of official publications of the AMA CPT Editorial Bodies. These references are publicly available and are cited here. 15 16 Additionally, the task force conducted direct correspondence with AMA CPT coding committee representatives.

### Assessment of Contemporary Coding Trends Among Active Surgeons

Fellow members of the American Neurotology Society (ANS) were queried via survey instruments distributed in conjunction with the ANS Fall Meeting held in Nashville, Tennessee on September 30, 2023. The 9-item questionnaire was distributed electronically 60 days prior, 30 days prior, and 7 days prior to the meeting. The audience was also given time to respond in person during the meeting in real time via QR code links to the instrument. At the time of final analysis, any duplicate responses were removed. A complete list of the survey items and responses is provided in Appendix A (available in online version only).

## Results

### Background Brief on CPT Codes and Regulating Bodies

The use of CPT codes began over 50 years ago with the purpose of facilitating a uniform language for coding medical services and procedures. CPT codes are also used in contemporary times for administrative management purposes, claims processing, guideline development, and performance metrics. Category I CPT codes are utilized for most surgical procedures and require linkage to appropriate International Classification of Diseases (ICD) codes. In the case of surgery for VS, CPT codes are linked to ICD-10 code D33.3 “benign neoplasm of cranial nerve.”

New CPT codes are developed through a multi-tiered committee system, including the AMA CPT Editorial Panel and affiliated CPT Advisory Panels (21 members, including 12 physicians nominated from specialty medical societies). When a new CPT code is formally adopted, it is then assigned a relative value unit (RVU) figure by CMS. The latter process governs financial compensation and is designed to reflect physician labor, material expenses, institutional resources, and nonclinical resources applied to a given procedure.

The related value marker work-RVU (wRVU), which more closely tracks technical, physical, and logistical costs incurred by individual providers, is derived from the RVU. This assignment of RVU/wRVU is overseen by Relative value system Update Committees (RUCs) which meet three times yearly. RUCs conduct surveys of specialty society members (QHPs) and use this information to recommend an accurate RVU value for the CPT code under review. This RVU recommendation is submitted to CMS. The RUC committee schedule is public and can be accessed from the AMA Web site. Ultimately, CMS makes all final decisions about actual reimbursement, regardless of RUC recommendations. Private payers typically structure reimbursement upon CMS valuations. Further discussion on RUCs and RVU/wRVU is beyond the scope of this investigation, but excellent review materials may be accessed. 17 18 19 20

Ultimately, CPT code creation is based upon a rigorous, evidence-based and open process. Overseeing entities are responsible for maintaining clinical validity, relevance, and timeliness of the CPT codes and periodically updating them to reflect contemporary innovation in medicine. CPT Editorial Panel meets three times yearly to coincide with the AMA RUC meetings.

Confusion over the correct application of CPT codes often occurs in two settings. The first is in the realm of new medical/surgical innovations and/or technological advancements. In such cases patient care may have no relevant procedure-specific CPT code assignment, thus requiring the development of new codes. The second setting is in the application of CPT codes to procedures involving more than one surgeon. Regarding the latter, CMS maintains the National Correct Coding Initiative (NCCI) program which oversees and regulates the use of multiple CPT codes in a given procedure (and in procedures with multiple coding surgeons). The NCCI regulation is designed to prevent improper payment of procedures that should not be submitted together (improperly unbundled) and is defined in the NCCI Policy Manual in the “Multiple Procedure Rule,” Medicare NCCI Policy Manual, Chapter 1. 21

Key takeaways from the NCCI Policy Manual include the following:

A. CPT codes will have decreasing values when more than one category I code is assigned to a single case. CMS will only pay “full price” for the highest-valued category I CPT code. The second most valued code is paid at 50%. All subsequent procedures are paid at 25%.
B. In a co-surgical procedure, a -62 modifier may be attached to the CPT code. However, each CPT code is assigned with a “co-surgeon indicator” on the use of -62 modifiers. The co-surgeon indicator levels are: 0 (co-surgeon not permitted), 1 (co-surgeon may be allowed if documentation supports), and 2 (co-surgeon permitted without additional documentation). When coding a -62 modifier of a CPT code, the reimbursement of the code is set at 125% of the RVU value, which is then divided equally between the co-surgeons.

### Regarding CPT Coding for CPA Tumor Surgery

Following the inaugural institution of CPT codes in the 1960s, the so-called legacy or “traditional” codes were developed for removal of VS. Over time however, many skull base surgeons found the traditional CPT codes to be lacking in their ability to capture the considerable variations in case complexity and operative time/effort associated with VS of different size, location, and phenotype. 7 10 22 This issue was further complicated by modern improvements in both operative technology and preoperative imaging (allowing earlier detection of tumors). At that time, most neurosurgical and neurotologic skull base surgeons agreed that the traditional CPT codes were not sufficient.

In the early 1990s, a prominent panel comprised of a neurotologist (John Leonetti, MD) and two neurosurgeons (Thomas Origitano, MD, PhD, and Byron Pevehouse, MD) was tasked by the North American Skull Base Society with developing a new set of open lateral skull base CPT codes. The panel began by officially determining that the existing traditional codes did not adequately describe the procedures and division of labor associated with most CPA tumors. Ultimately this panel generated a list of 98 novel CPT codes, later revised by CMS to 28 codes that are currently in use. These “skull base” codes were comprised of “approach codes” and paired “resection codes.” Notably, following the creation of these newer split codes, the legacy/traditional codes were not retired.

Both coding strategies may be applied to tumors of the CPA, including VS, with no terminology or descriptors in the code sets to imply limited use. The proper application of the code sets does differ, however. A detailed breakdown of the traditional/legacy code set is provided in Table 1 . The skull base code sets are demonstrated in Table 2 . The most important difference between the code sets comes in the form of the NCCI multiple procedure policy and Medicare Physician Fee Schedule Database Indicators (MPFSDI) on co-surgical coding. The traditional codes combine approach/resection into a singular code which is shared between the co-surgeons. As such, they are associated with an Indicator of 2. Use of a -62 modifier is allowed without supporting documentation. The skull base codes entail use of both an approach code and a resection code and are associated with an MPFSDI Indicator of 1. Supporting documentation is required to establish medical necessity of -62. Skull base procedure/billing codes must match between the co-surgeons as does supporting documentation. Alternatively, each surgeon may submit one approach and one resection code, respectively, without a -62 modifier.

**Table 1:** Table demonstrating the Medicare Physician Fee Schedule Database (MPFSDB) Indicator 2 codes used for tumors of the cerebellar pontine angle. Also known as the traditional/legacy codes or the combined approach/resection codes. Additional information on descriptors, use of modifiers, and use of ancillary codes are also provided

Combined approach/resection codes (traditional/legacy) MPFSDB Indicator 2 a			
CPT code	Practical descriptor/Application	Use of modifier(s)	Use of ancillary codes c
61520 - Infratentorial or posterior fossa craniectomy for excision of cerebellopontine angle tumor	Retrosigmoid approach for removal of CPA tumor	Indicator 2 b —OK to use -62 modifier without supporting documentation. Co-surgeons split the code value.	Fat/fascia graft— *15769* 15770 Use of microscope— *69990* Use of image guidance 61781 Intraoperative neuromonitoring (IONM) d 95940: in-person 95941: remote G0453: remote
61526– Craniectomy, bone flap craniotomy, transtemporal (mastoid) for excision of CPA tumor	Translabyrinthine approach for removal of CPA tumor		
61530– Combined transtemporal and middle or posterior fossa excision of CPA tumor	Middle fossa approach for removal of CPA tumor		

a MPFSDB - Medicare Physician Fee Schedule Database Indicators.

b Indicator of 2—the payment rule for two surgeons apply. -62 is allowed without supporting documentation. Procedure/Billing codes must match.

c Use of ancillary codes may be applied with supporting documentation. NCCI coding rules apply with additional codes following the category I code. Cannot apply -62 modifier to ancillary codes. Only one co-surgeon may code.

d CPT codes for intraoperative neuromonitoring (IONM). Coding depends on institution preference and type of service rendered, as follows:

**95940:** Continuous intraoperative neurophysiology monitoring in the operating room, one-on-one monitoring requiring personal attendance, each 15 minutes. Cannot be billed if the service is 7 minutes or less.

**95941:** Continuous intraoperative neurophysiology monitoring, from outside the operating room (remote or nearby) or for monitoring of more than one case while in the operating room, per hour. Bill for each hour of monitoring performed by a remote professional, potentially supervising multiple patients or procedures. Cannot be billed for 30 minutes or less.

**G0453:** Continuous intraoperative neurophysiology monitoring, from outside the operating room (remote or nearby), per patient (attention directed exclusively to one patient), each 15 minutes. Similar to 95941 but specifically for the remote, 15-minute interval for a single patient.

**Table 2:** Table demonstrating the Medicare Physician Fee Schedule Database (MPFSDB) Indicator 1 codes used for tumors of the cerebellar pontine angle. Also known as the skull base codes or the independent approach and resection codes. Additional information on descriptors, use of modifiers, and use of ancillary codes are also provided

*Approach* and *resection* codes (skull base) MPFSDB Indicator 1 a			
CPT code	Practical descriptor/Application	Use of modifier(s)	Use of ancillary codes
**Tumor approach codes**			
61595 - Transtemporal approach to the posterior cranial fossa, jugular foramen, or midline skull base, including mastoidectomy, decompression of sigmoid sinus and/or facial nerve, with or without mobilization	Retrosigmoid or translabyrinthine approach for CPA tumor	Indicator 1 a —Not recommended for split modifier. -62 only with supporting and matching documentation	Fat/fascia graft— *15769* 15770 Use of microscope— *69990* Use of image guidance 61781 Intraoperative neuromonitoring (IONM) c 95940: in-person 95941: remote G0453: remote
61596 - Transcochlear approach to the posterior cranial fossa, jugular foramen, or midline skull base including labyrinthectomy, decompression, with or without mobilization of facial nerve and/or petrous carotid artery	Translabyrinthine/Transcochlear/ Transotic approach for CPA tumor		
61590/61591 - Infratemporal approach to the middle cranial fossa (internal auditory meatus, petrous apex, tentorium, cavernous sinus, parasellar area, infratemporal fossa) including mastoidectomy, resection of sigmoid sinus, with or without decompression and/or mobilization of the contents of the auditory canal or petrous carotid artery	Middle fossa approach for CPA tumor		
**Tumor resection codes**			
61616 - Resection or excision of a neoplastic, vascular, or infectious lesion of base of posterior cranial fossa, jugular foramen, foramen magnum, or C1–C3 vertebral bodies, intradural, including dural repair, with or without graft	Tumor resection code for posterior fossa tumors (used with 61595, 61596). Could be considered for middle fossa approach if tumor extends to the posterior fossa.	Indicator 1 a —Not recommended for split modifier. -62 only with supporting and matching documentation	
61606 - Resection or excision of neoplastic, vascular, or infectious lesion of infratemporal fossa, parapharyngeal space, petrous apex; intradural, including dural repair, with or without graft	Tumor resection code for middle fossa and infratemporal fossa tumors (used with 61590/61591). Tumor confined to temporal bone structures/internal auditory canal.		

a MPFSDB - Medicare Physician Fee Schedule Database Indicators. Indicator of 1—supporting documentation is required to establish medical necessity of -62. Procedure/Billing codes must match between surgeons as does both sets of documentation. More likely to face insurance review/adjustment if -62 is utilized. Alternatively, each surgeon may submit one approach and one resection code, respectively.

b Use of ancillary codes may be applied with supporting documentation. NCCI coding rules apply with additional codes following the category I code. Cannot apply -62 modifier to ancillary codes. Only one co-surgeon may code.

c Depending on institution preference, a separate service may perform and bill for this activity. See footnote d, Table1 .

### Assessment of Contemporary Coding Trends

A 9-item questionnaire was distributed to active fellow members of the ANS at the 2023 Fall Conference hosted in Nashville, TN. A total of 161 responses were received. The estimated response rate among conference attendees was 36% (total attendance of active fellows that year was 447). The overall ANS membership (active fellows) in 2023 was estimated to be 520, indicating that the survey response rate society-wide was 31%.

The majority of responders (84%) were aware of their/their institution's coding strategy. About 37% of responders noted they don't code for themselves but were aware of the coding strategy utilized. About 14% were unsure of codes used for CPA tumors at their institution.

The practice profile distribution among responders is shown in Fig. 1 . Responders were representative of academic, institutional, and private practice settings. The majority of responders indicated they were experienced with CPA tumor surgery and coding ( Fig. 2 ). Out of all responders 77% indicated that they perform ≥6 AN surgeries per year. About 80% of all responders were ≥7 years out of fellowship training.

**Fig. 1:**
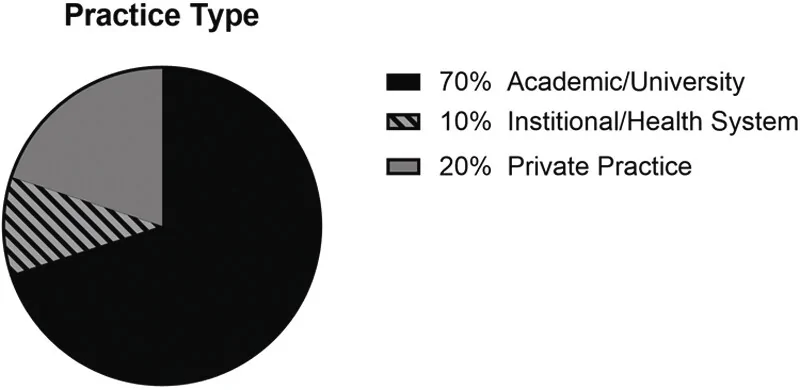
Practice profile distribution among responding surgeons. Pie graph demonstrating practice type among responding surgeons. Response rate on this instrument was estimated to be 31% of the American Neurotology Society membership in 2023 (data not shown).

**Fig. 2:**
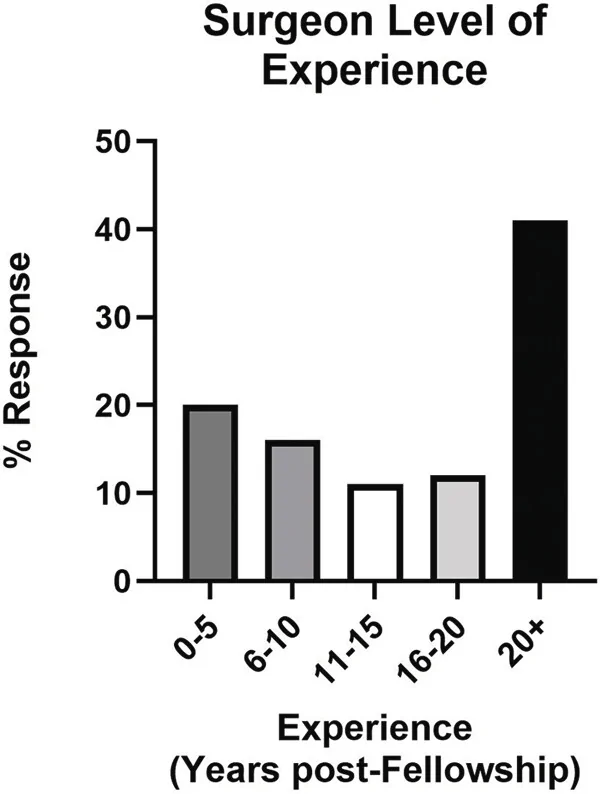
Post-fellowship experience with cerebellopontine angle (CPA) tumor surgery. Column graph depicting the experience of survey responders in terms of number of years in practice following completion of certified fellowship training. The y-axis indicates percentage of all respondents.

Regarding the prevalence of coding strategy according to approach, the skull base codes were the most commonly used for translabyrinthine ( Fig. 3 ), retrosigmoid ( Fig. 4 ), and middle fossa approaches to CPA tumors and posterior cranial fossa ( Fig. 5 ). Among those responders using the skull base codes, roughly 60% coded both the approach and resection codes with a -62 modifier attached to each. The remainder indicated that they only utilize the approach code without a modifier. For each approach, 40% of responders indicated they utilized the traditional codes for these procedures.

**Fig. 3:**
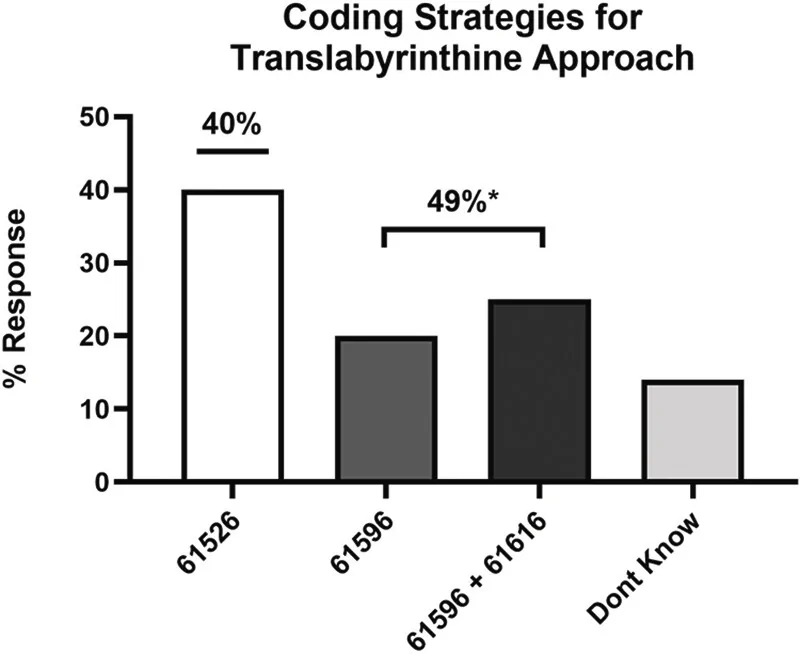
Coding strategies for translabyrinthine approach and resection of cerebellopontine angle tumors. Column graph depicting survey responses on coding strategies used for the translabyrinthine approach. 61526 is a traditional/combined code. A total of 49% of respondents indicated use of the skull base approach code (61596), either in isolation (second column; neurosurgeon codes resection with no modifier) or paired with the tumor resection code (third column; 61616) (*). The latter requires use of -62 modifiers on both codes with adequate supporting documentation. The y-axis indicates percentage of all respondents.

**Fig. 4:**
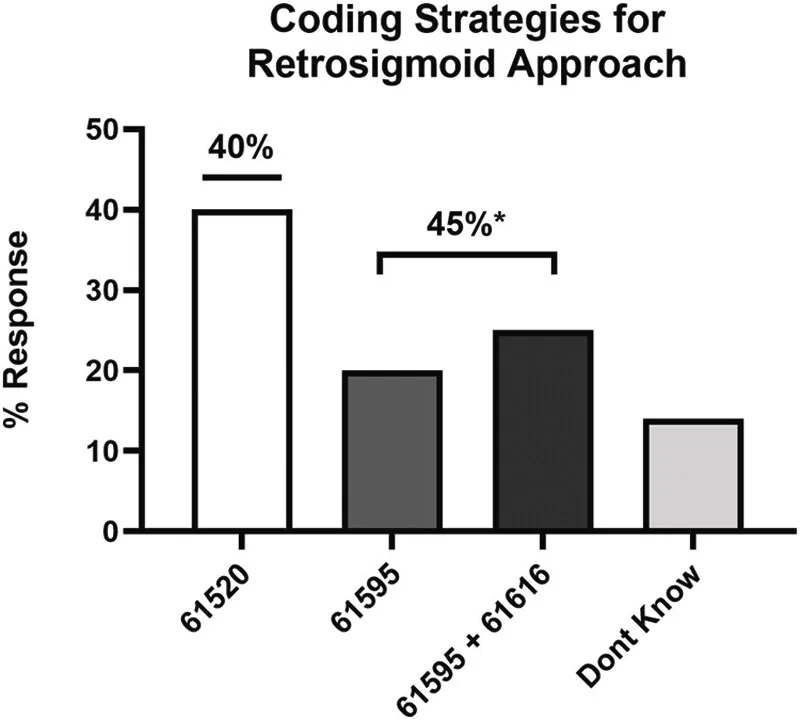
Coding strategies for retrosigmoid approach and resection of cerebellopontine angle tumors. Column graph depicting survey responses on coding strategies used for the retrosigmoid approach. 61520 is a traditional/combined code. A total of 45% of respondents indicated use of the skull base approach code (61596), either in isolation (second column; neurosurgeon codes resection with no modifier) or paired with the tumor resection code (third column; 61616) (*). The latter requires use of -62 modifiers on both codes with adequate supporting documentation. The y-axis indicates percentage of all respondents.

**Fig. 5:**
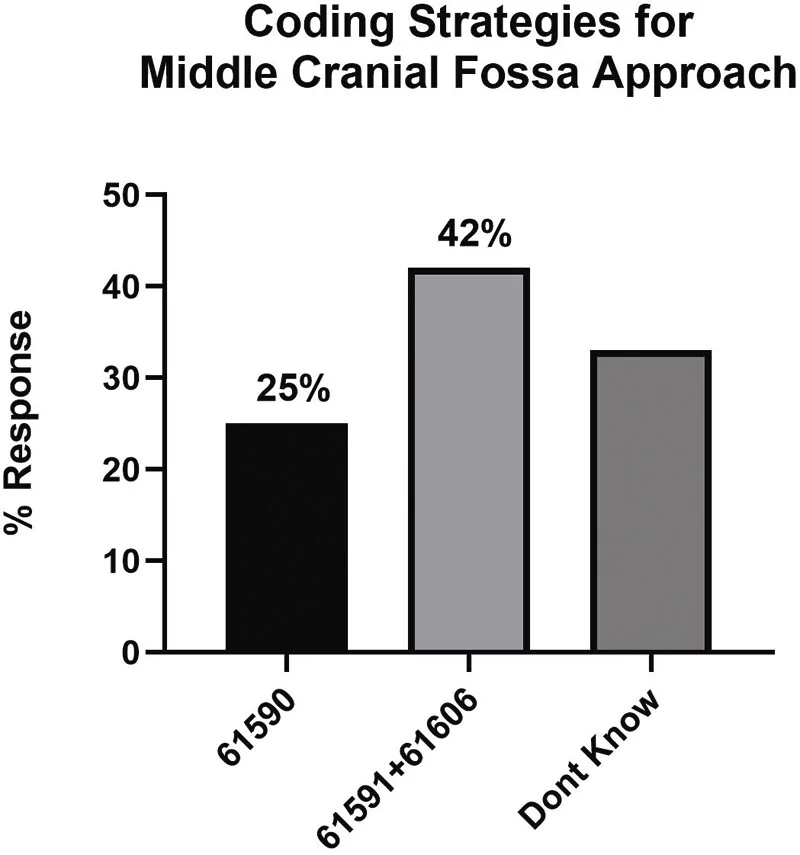
Coding strategies for middle cranial fossa approach and resection of cerebellopontine angle tumors. Column graph depicting survey responses on coding strategies used for the middle cranial fossa approach. In preliminary assessments of coding prior to our survey instrument, no responders indicated use of the traditional/combined code 61530 (data not shown). All responders who were aware of their coding strategy indicated use of the skull base approach code (61590 or 61591), either in isolation (first column; neurosurgeon codes tumor resection with no modifier) or paired with the tumor resection code (second column; 61606). The latter requires use of -62 modifiers on both codes with adequate supporting documentation. The y-axis indicates percentage of all respondents.

## Discussion

Currently, two CPT coding strategies exist for surgical management of CPA tumors (particularly VS). As of this writing, the AMA CPT Editorial Panel and CMS formally recognize both the traditional and skull base CPT coding schemes as evidenced in official publications. This was confirmed by our task force via direct correspondence with panel representatives.

Based upon our investigations, it appears that use of the skull base codes is slightly more common than use of the traditional codes among members of the ANS. As previously mentioned, this was reflective of the limited data available in the literature. It should be noted that the data presented here is limited to ANS (neurotology) membership only. However, a publication by Bi et al in 2019 did report on coding strategy prevalence among prominent neurosurgical institutions with similar findings. This publication also looked at CPT codes used in retrospective research on VS, which may further indicate institutional trends/preferences in coding on a broader scale. 8

Due to the plurality of coding strategies currently available, there has recently been debate regarding how to “correctly” code CPA surgeries. 9 10 13 14 22 Based on the available evidence, this task force would conclude that neither the skull base codes nor the traditional codes should be considered “correct.” It is more important that providers educate themselves on the nature of CPT coding as well as the nuances of how each coding set is properly implemented.

Aside from this, one specific point to address is the use of skull base approach codes for translabyrinthine and other trans-petrosal approaches. In this case, both 61595 and 61596 have supporting verbiage in their descriptors such that they might apply to a spectrum of trans-petrosal approaches ( Table 2 ). Within our survey instrument, and as part of this discussion, 61596 was utilized to represent the translabyrinthine approach (being the only descriptor containing “labyrinthectomy”). This was based on our memberships' predilection to utilize 61595 for retrosigmoid approaches and 61596 for translabyrinthine/transcochlear approaches. However, the 61596 descriptor also contains the word “transcochlear.” This should not preclude use in translabyrinthine approaches, however. This assertion is based upon the fact that the labyrinthine organs and cochlea are anatomically connected to each other, and are housed together within a single bony structure (the otic capsule). Thus, labyrinthectomy (trans-labyrinthine) does technically/anatomically entail a partial transcochlear exposure in all cases. Regardless, providers utilizing this coding strategy should employ detailed supportive documentation based on the surgical strategy employed.

To that end, it is important to point out the code sets do differ significantly with respect to co-surgery indicators, the use of -62 modifiers, and the degree of supporting documentation required ( Tables 1 and 2 ). The choice of which coding strategy to adopt should be based on institutional preferences, the surgical strategy employed, and how detailed the supporting documentation is. The skull base code set demands the most detailed documentation, especially in cases where modifiers are employed. Additionally, neurotologists/otologists and neurosurgeons should engage in discussion on coding strategy to ensure each specialist's codes and supporting documentation properly align. This discussion would also ideally include each group's respective certified coders. Strategic alignment in this manner is a must to avoid denials/adjustments by payers.

Finally, resident and fellow education on proper coding practice is impacted by a plural coding situation. Trainees should educate themselves on both the traditional and skull base code sets, including how they are properly applied. Given that ACGME minimum-case requirements in training utilize both code sets (and in unequal fashion), proper case logging does demand some familiarity with this topic. Directors of skull base surgical fellowships should consider exposure of their trainees to these coding concepts early in their curriculum.

This study does have weaknesses. Notably, the task force was not purposed to explore billing and/or collections associated with a given code set. As such, extrapolation regarding fiscal benefit of one code set over another was not feasible. Second, our survey instrument indicated that 11% of providers did not code for themselves *and* were uncertain which code type is used in their approaches to CPA tumors. Finally, data representing neurosurgical coding practice are limited at this time.

## Conclusion

Two coding strategies for the surgical management of CPA tumors (including VS) exist. The regulatory bodies of the AMA CPT Editorial Panel, the relevant CPT Advisory Panels, and CMS currently support both coding strategies. Data from this investigation suggest surgeons from all practice types are utilizing both code sets at roughly equal rates. It is important that surgeons performing CPA tumor surgery understand the difference in the code sets and how they are applied. It is important for institutions to align regarding co-surgical billing, especially with use of the skull base approach/resection codes.
