# Supplementary material for: Contemporary Use of Common Procedural Terminology (CPT) Codes for Surgical Management of Cerebellopontine Angle Tumors
Source: J Neurol Surg B Skull Base. 2026 May 11;87(6):634–40. doi: 10.1055/a-2790-6085 (PMC13730694; doi:10.1055/a-2790-6085)
Supplement: Supplementary file 1 [file 10-1055-a-2790-6085_28735311.pdf]

Thieme

Supplementary Material
Appendix A

Survey Instrument Distributed to Active Surgeon Members of the American Neurotology Society, 2023 Fall Meeting. Nashville, TN. Percentages in parentheses indicate percent of respondents.

1. How long have you been in practice?

- 0–5 years (20%)
- 6–10 years (16%)
- 11–15 years (11%)
- 16–20 years (12%)
- 21+ years (41%)

2. In what environment is your practice located?

- Academic (70%)
- Private practice (10%)
- Institution/Health system (20%)
- Other (0%)

3. How many acoustic neuroma tumors do you operate on per year?

- 0–5 (27%)
- 6–15 (35%)
- 16–30 (20%)
- >30 (18%)

4. What metric is primarily used to determine your compensation?

- Fixed salary (22%)
- Collections (9%)
- Relative value unit; RVU (11%)
- Fixed salary with collection-based incentives (32%)
- Fixed salary with RVU-based incentives (26%)
- Other (please specify) (0%)

5. Who performs coding for your procedures?

- You (33%)
- A non-healthcare provider member of your practice or health system (63%)
- Physician assistant/nurse practitioner in your practice or health system (0%)
- Resident or fellow in your practice or health system (0%)

- Other (please specify) (4%)

6. Who performs billing for your procedures?

- You (18%)
- A non-health care provider member of your practice or health system (80%)
- Physician assistant/nurse practitioner in your practice or health system (0%)
- Other (please specify) (2%)

7. When you perform a Translab approach for an acoustic neuroma, what CPT code do you use?

- 61526 (40%)
- 61595/96 (17%)
- 61595/96 + 61616 (32%)
- Other/Don’t know (11%)

8. When coding YOUR involvement in a Retrosig approach for an acoustic neuroma, what CPT code do you use?

- 61520 (40%)
- 61595 (20%)
- 61595 + 61616 (25%)
- Other/Don’t know (14%)

9. When coding YOUR involvement in a middle fossa approach for an acoustic neuroma, what CPT code do you use?

- 61590 (25%)
- 61591 + 61606 (42%)
- Other/Don’t know (33%)
